# Accuracy and precision of myocardial T_1_mapping with MOLLI and ShMOLLI at 1.5T and 3.0T: a phantom study

**DOI:** 10.1186/1532-429X-16-S1-P54

**Published:** 2014-01-16

**Authors:** Claire Falque, Frank Kober, Monique Bernard, Alexis Jacquier

**Affiliations:** 1 Radiology, APHM, Marseille, France; 2 CRMBM, Aix Marseille Université UMR CNRS n° 7339, Marseille, France

## Background

Previous studies have demonstrated that an increase in extracellular volume measured on MRI is directly proportionate to myocardial fibrosis. Myocardial extracellular volume is proportionate to partition coefficient (λ) and extracellular volume = λ × (1-hematocrit). λ can be measured by assessing the T1 relaxation time before and after injection of contrast medium. The goal of this study is to assess the accuracy and precision of several T1 mapping sequences using a phantom at 1.5 and 3.0T.

## Methods

Experiments were performed on two magnets; a 1.5T magnet (Avanto, Siemens) and on a 3.0T magnet (Verio, Siemens) with a 32 phased array cardiovascular coil. phantoms were built using 15 separate tubes to produce similar T1 and T2 values of myocardium and blood before and after gadolinium administration. All sequences were tested with an ECG simulation at heart rates 40, 60, 90, 120. To assess the standard T1 value for each tube, we used a tubo spin echo inversion-recovery sequence (TR/TE = 13000/18 ms, with 17 inversion times between 30-9000 ms turbo factor = 7). The T1 value was assessed using an ECG gated single shot modified Look Locker inversion recovery (MOLLI) and several different schemes of Short MOLLI: at 1.5T (α:35°, 5-2 and 4-3-2 sampling schemes; with a pause of 3 HB) and at 3.0T (α:35° and 20°; 5-2 and 4-3-2 sampling schemes; pause: 3 and 4 HB). We calculated errors in the MOLLI T1 estimation according to Error T1 (%) = ((T1MOLLI-T1reference)/T1reference)x100. We assessed the effect of T2 value, T1 value, heart rate and MOLLI schemes on T1 errors.

## Results

**Figure 1:**
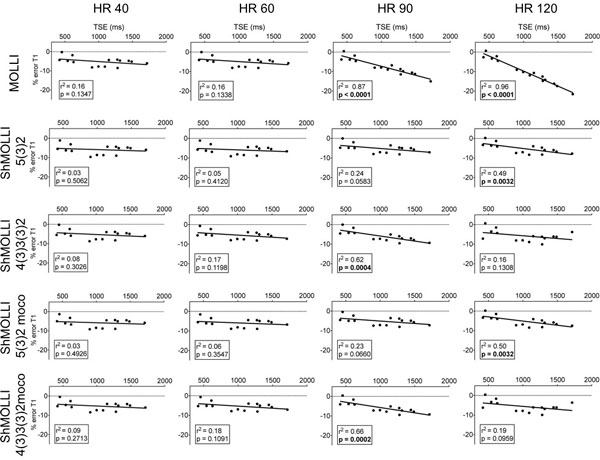
**Showing the linear regression between % T1 error of the different sequences compared to reference T1 value at each heart rate tested at 1.5T**.

**Figure 2:**
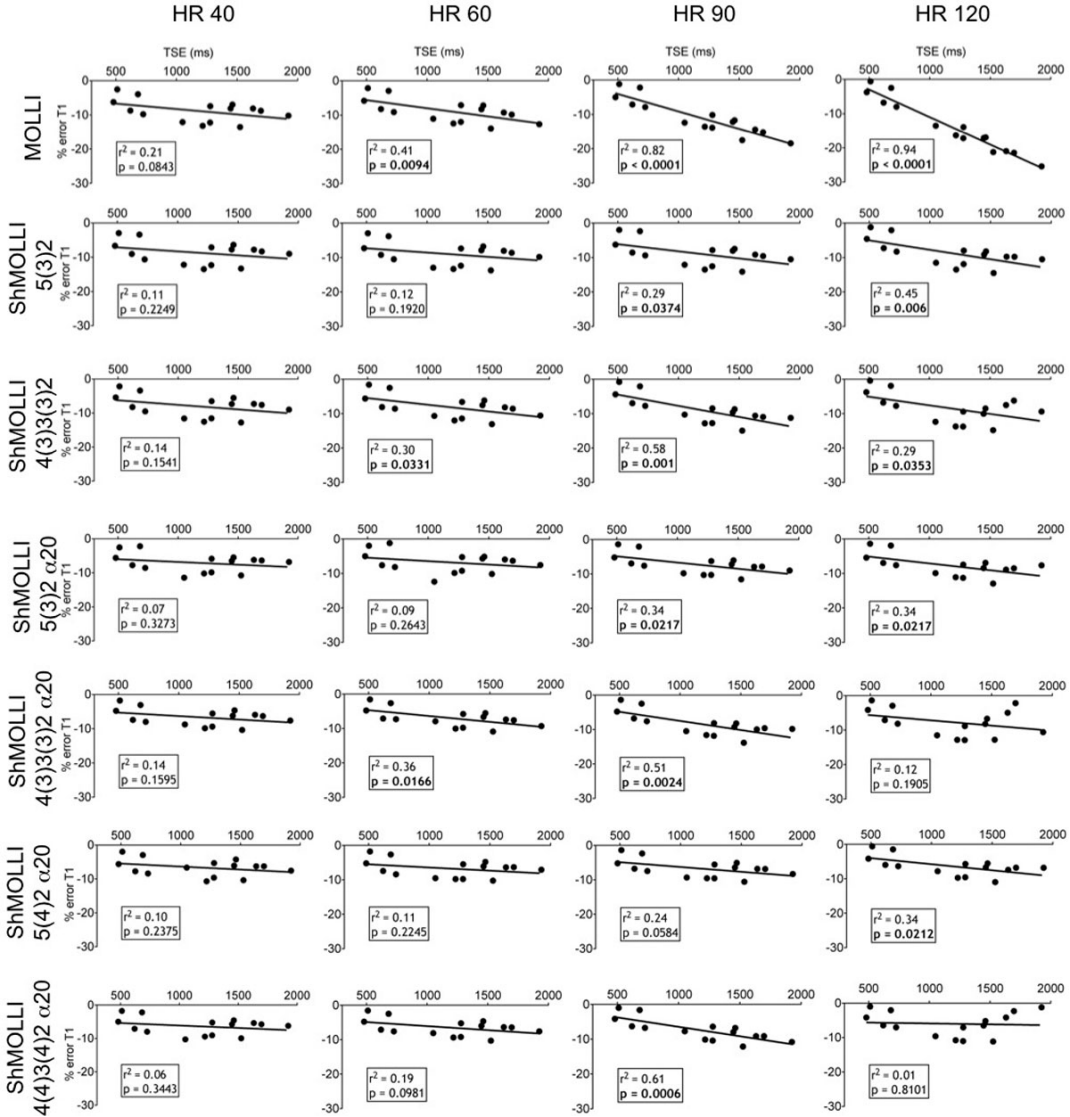
**Showing the linear regression between % T1 error of the different sequences compared to reference T1 value at each heart rate tested at 3.0T**.

At 1.5 and 3.0T, the under estimation of T1 with MOLLI sequence was important, highly dependent on heart rate and increased significantly from 40 to 120 bpm. Using ShMOLLI sequences, the effect of heart rates was lower than 1% at 1.5T (Figure 1), and was not dependent on heart rate at 3.0T (Figure 2). The underestimation of T1 was present in shorter T2 phantoms and less in longer T2 phantoms. The effect of T2 on T1 error was predominant at low heart rates. Underestimation of T1 was predominant in higher T1 and at higher heart rate. We measured a significant relationship between the T1 value and % of T1 error. At 3.0T, acquisitions with a flip angle at 20° showed less T1 underestimation compared with 35°. The addition of one heart beat pause showed less underestimation for longer T1 with the 5-2 schemes but did not change the results significantly for short T1 values.

## Conclusions

ShMOLLI sequences provide an accurate assessment of T1 at 1.5T and 3.0T.

## Funding

This study was supported by the PHRC 2011-A00887-34.

